# Supplementary material for: Wide range of possible trajectories of North Atlantic climate in a warming world
Source: Nat Commun. 2024 May 17;15:4221. doi: 10.1038/s41467-024-48401-2 (PMC11101628; doi:10.1038/s41467-024-48401-2)
Supplement: Supplementary file 1 — Supplementary Information [file 41467_2024_48401_MOESM1_ESM.pdf]

Supplementary Information for: "Wide range of possible trajectories of North Atlantic climate in a warming world"

Qinxue Gu, Melissa Gervais, Gokhan Danabasoglu, Who M. Kim
Frederic Castruccio, Elizabeth Maroon, Shang-Ping Xie

1 Supplementary Figures

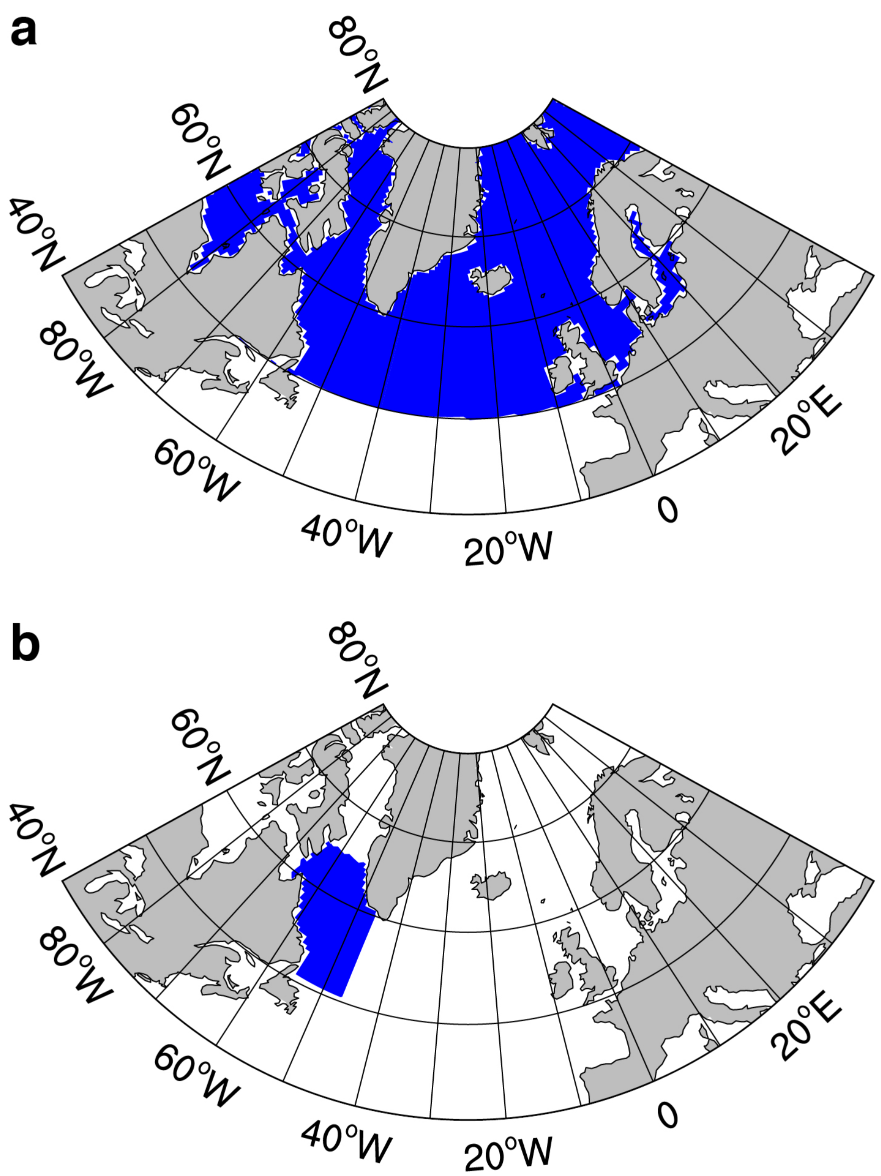

Supplementary Fig. 1. The domains used for area-weighted average calculation. **a**, northern North Atlantic. **b**, Labrador Sea.

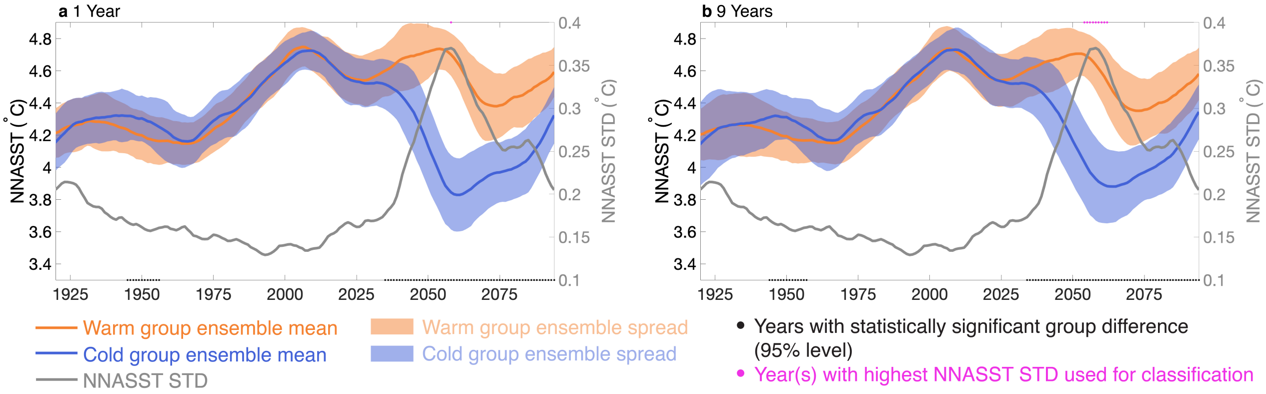

Supplementary Fig. 2. Sensitivity test on the classification criteria of the warm and cold groups. **a**, same as Fig. 1a, but the warm and cold groups are classified based on their winter northern North Atlantic SST (NNASST) during the year with the highest NNASST standard deviation. Ensemble members are assigned to the warm (cold) group if their NNASST index is greater (smaller) than or equal to the ensemble mean plus (minus) one standard deviation of the NNASST in the CESM2 piControl simulation during that year. **b**, same as **a**, but the warm and cold groups are classified based on any year of the 9 years with the highest NNASST standard deviation.

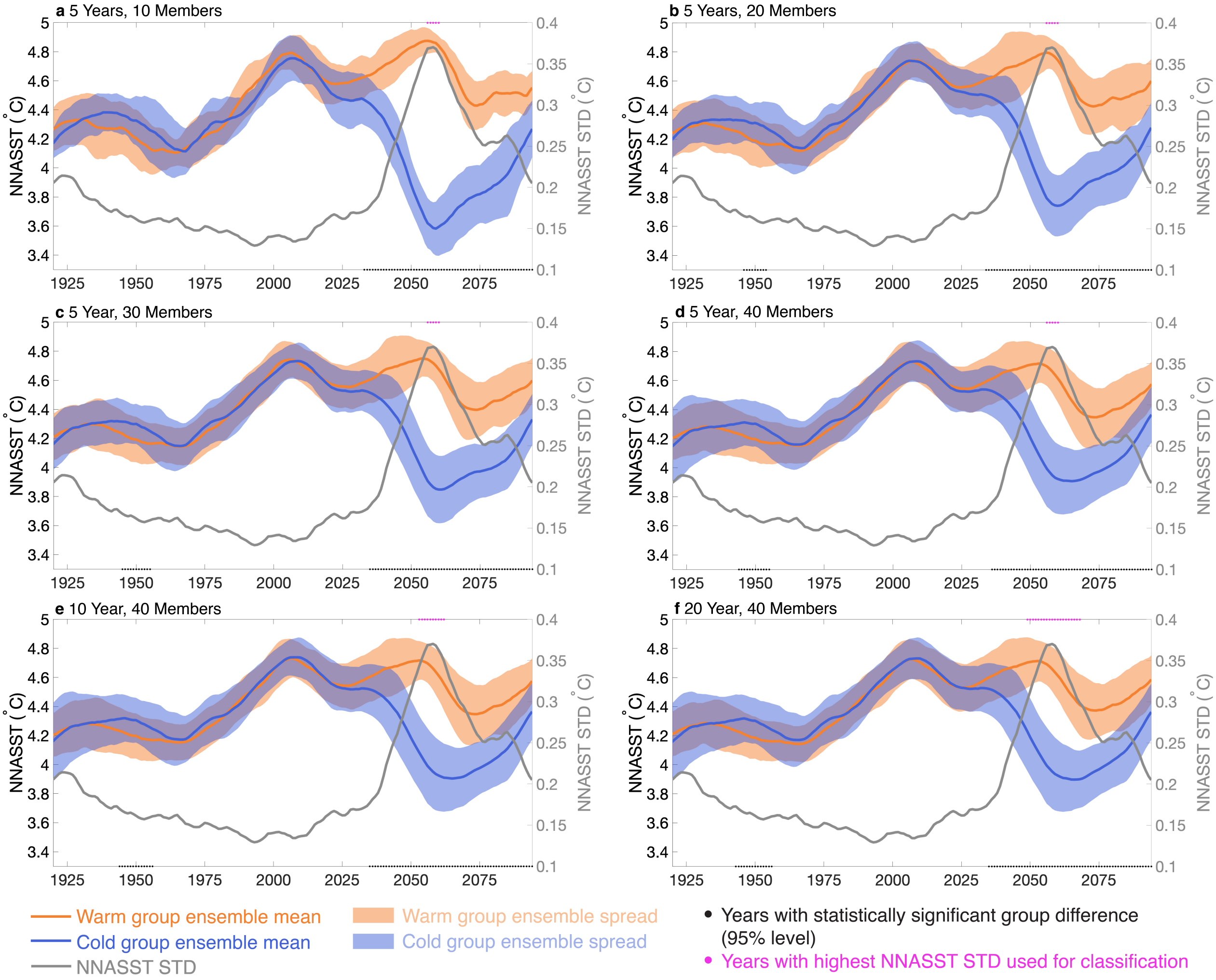

Supplementary Fig. 3. Sensitivity test on the classification criteria of the warm and cold groups. Same as Fig. 1a, but an equal number of ensemble members are classified into the warm and cold groups based on the average northern North Atlantic SST (NNASST) index over a number of years with the highest NNASST standard deviation. **a**, top and bottom 10 members based on the 5 years with the highest NNASST standard deviation. **b**, top and bottom 20 members based on the 5 years with the highest NNASST standard deviation. **c**, top and bottom 30 members based on the 5 years with the highest NNASST standard deviation. **d**, top and bottom 40 members based on the 5 years with the highest NNASST standard deviation. **e**, top and bottom 40 members based on the 10 years with the highest NNASST standard deviation. **f**, top and bottom 40 members based on the 20 years with the highest NNASST standard deviation.

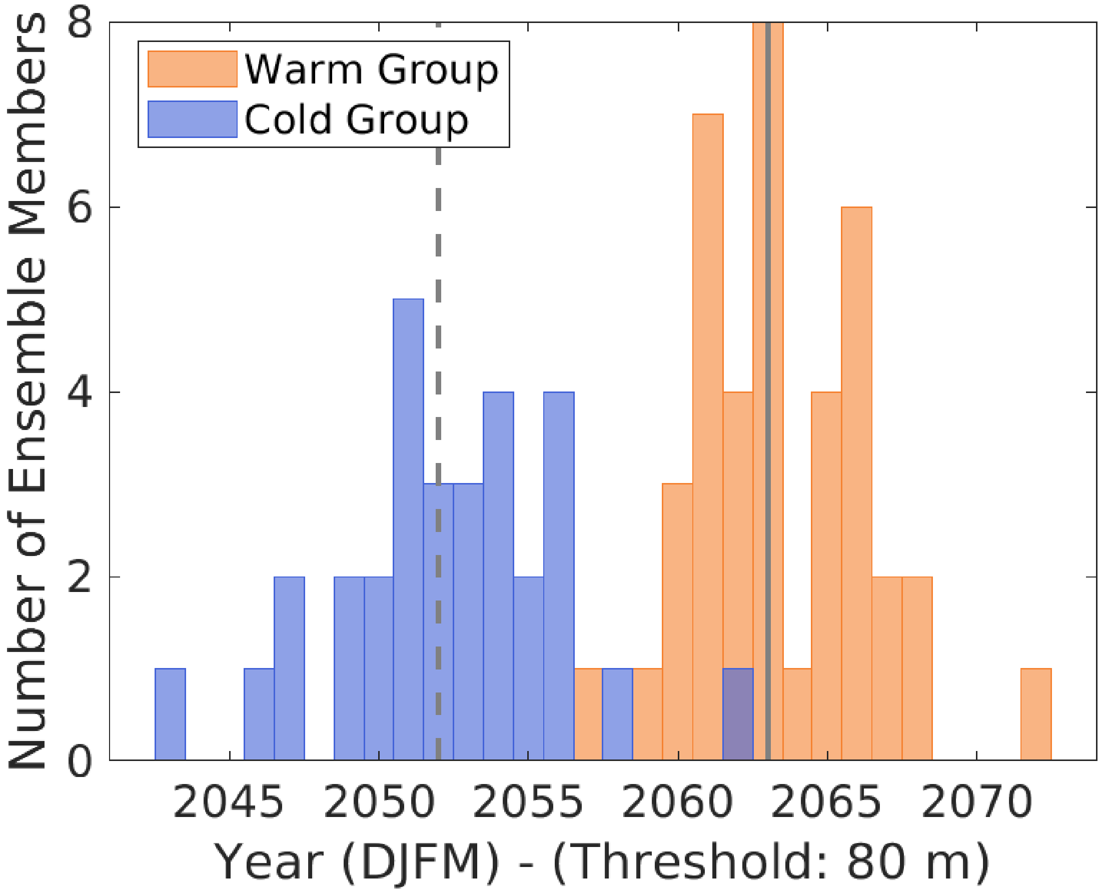

Supplementary Fig. 4. Different timing of deep convection shutdown between groups. Histograms of the year when each ensemble member in the warm (orange) and cold (blue) groups reaches an 80-m threshold of Labrador Sea mixed layer depth (denoted by the dashed black line in Fig. 1b). The median for the warm (2063) and cold (2052) groups is denoted by the solid and dashed vertical grey lines, respectively. This difference in timing is not sensitive to the threshold we choose.

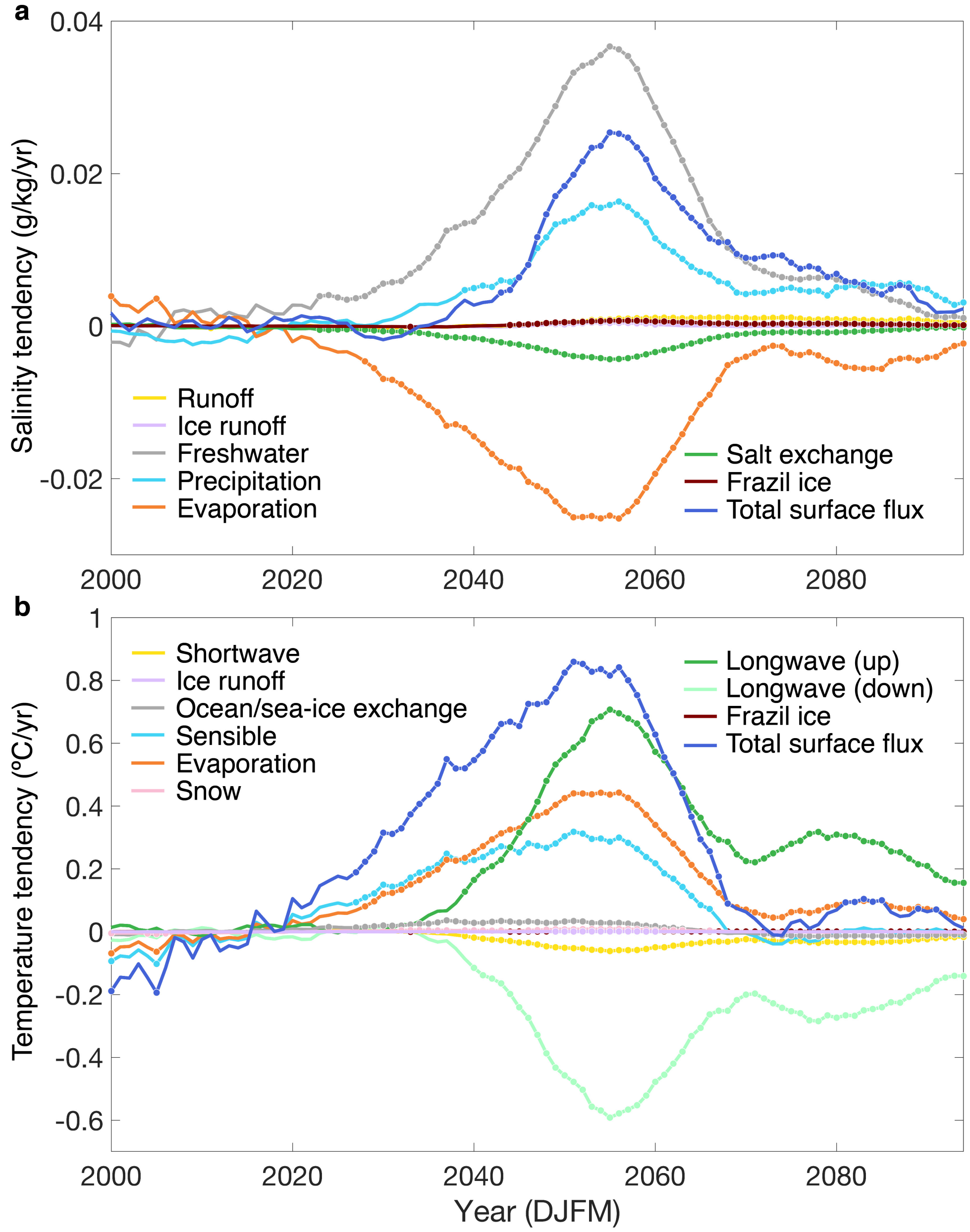

Supplementary Fig. 5. Salinity and heat budget analysis in the upper 295 m of the Labrador Sea: Decomposition of the surface flux. **a**, differences between the cold and warm groups (cold - warm) in total surface salinity flux (g/kg/yr; blue), as well as contributions to the surface flux from runoff (yellow), ice runoff (purple), freshwater fluxes between the ocean and sea-ice models due to processes such as sea ice and snow melting (grey), precipitation (cyan), evaporation (orange), salt exchange between the ocean and sea-ice models due to the salinity of sea ice (dark green), and frazil ice formation (maroon). **b**, differences between the cold and warm groups (cold - warm) in total surface temperature flux (°C/yr; blue), as well as contributions to the surface flux from shortwave radiation (yellow), ice runoff (purple), heat flux exchange between the ocean and sea-ice models (grey), sensible heat flux (cyan), evaporation (orange), snow (pink), upwelling longwave radiation (dark green), downwelling longwave radiation (light green), and frazil ice formation (maroon).

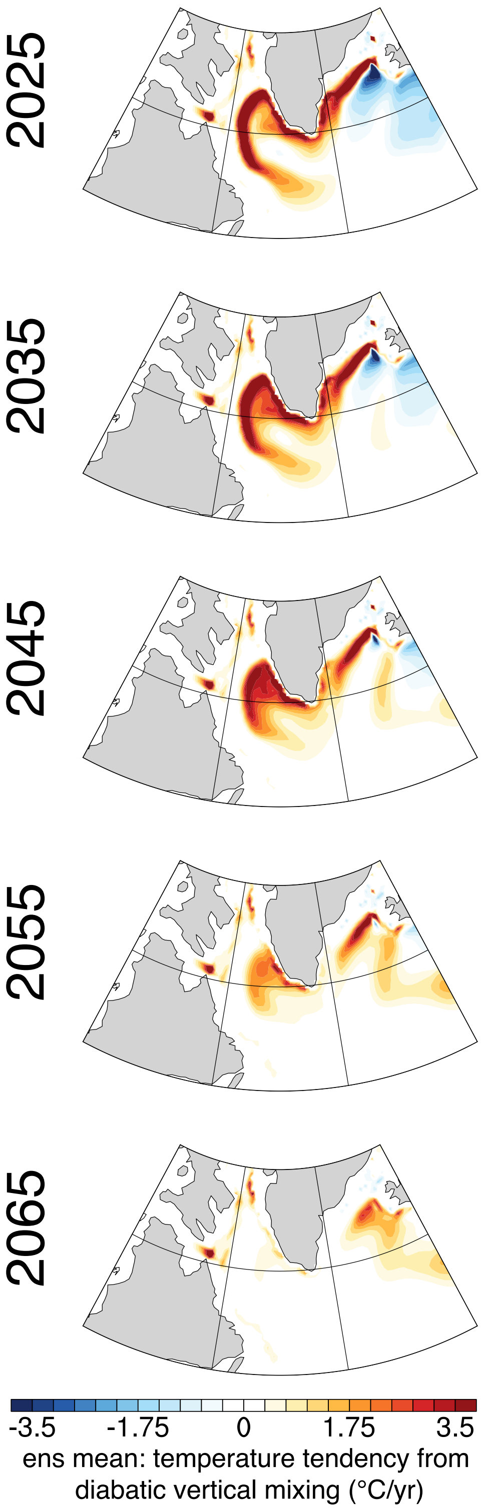

Supplementary Fig. 6. **100-member ensemble mean lowpass-filtered December-January-February-March temperature tendency contributed from diabatic vertical mixing for the upper 295 m (°C/yr).**

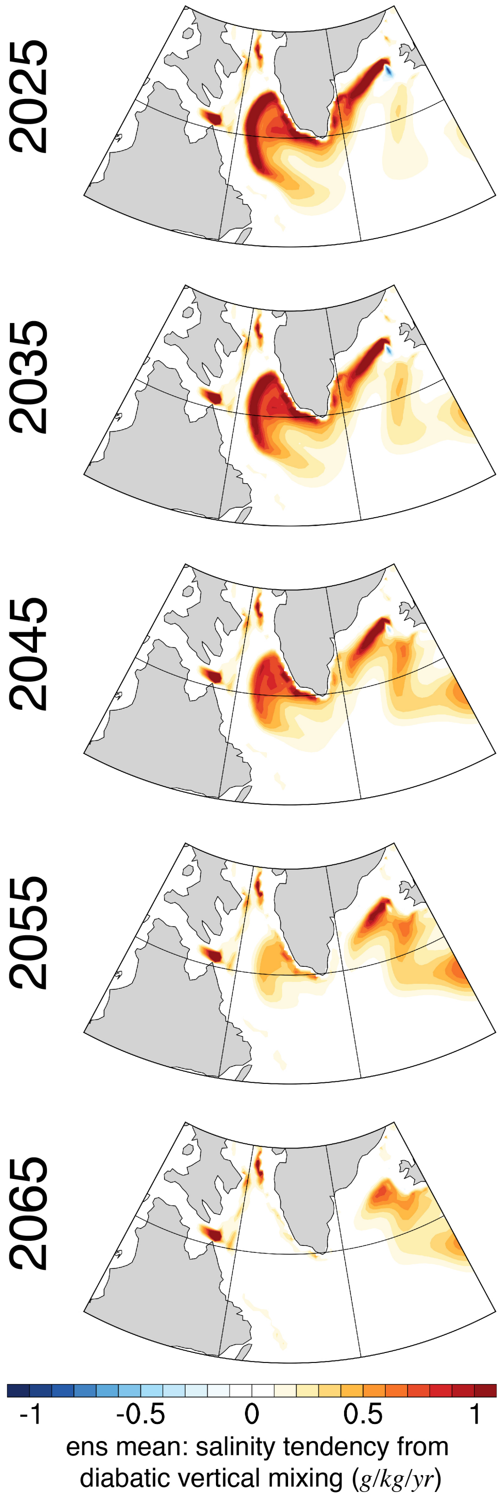

Supplementary Fig. 7. 100-member ensemble mean lowpass-filtered December-January-February-March salinity tendency contributed from diabatic vertical mixing for the upper 295 m (g/kg/yr).
