# Supplementary material for: Wide range of possible trajectories of North Atlantic climate in a warming world
Source: Nat Commun. 2024 May 17;15:4221. doi: 10.1038/s41467-024-48401-2 (PMC11101628; doi:10.1038/s41467-024-48401-2)
Supplement: Supplementary file 3 — Description of Additional Supplementary Files [file 41467_2024_48401_MOESM3_ESM.pdf]

Description of Additional Supplementary Files

File Name: Supplementary Movie 1
Description: Standard deviation (STD) of lowpass-filtered December-January-February-March (DJFM) North Atlantic sea surface temperature (SST; °C) across the 100 ensemble members.
